# Evaluation of Mechanical Properties of Porous Chitosan/Gelatin/Polycaprolactone Bone Scaffold Prepared by Microwave Foaming Method

**DOI:** 10.3390/polym14214668

**Published:** 2022-11-02

**Authors:** Shihan Wulin, Bing-Chiuan Shiu, Qian-Yu Yuan, He-qin Zhangjian, Jia-Horng Lin, Ching-Wen Lou

**Affiliations:** 1 College of Material and Chemical Engineering, Minjiang University, Fuzhou 350108, China; 2 Innovation Platform of Intelligent and Energy-Saving Textiles, School of Textile Science and Engineering, Tiangong University, Tianjin 300387, China; 3 Advanced Medical Care and Protection Technology Research Center, Department of Fiber and Composite Materials, Feng Chia University, Taichung 407802, Taiwan; 4 Advanced Medical Care and Protection Technology Research Center, College of Textile and Clothing, Qingdao University, Qingdao 266071, China; 5 Department of Medical Research, China Medical University Hospital, China Medical University, Taichung 40402, Taiwan; 6 Department of Bioinformatics and Medical Engineering, Asia University, Taichung 41354, Taiwan; 7 Fujian Key Laboratory of Novel Functional Fibers and Materials, Minjiang University, Fuzhou 350108, China

**Keywords:** chitosan, gelatin, mineral, montmorillonite, zinc oxide, titanium dioxide, microwave method, bone scaffold

## Abstract

Bone tissue may suffer from bone injury and bone defects due to accidents or diseases. Since the demand for autologous bone and allograft tissue far exceeds the supply, bone scaffolds have taken the lead. The use of bone scaffolds is one of the measures to help heal or regenerate bone tissue. Therefore, a new bone scaffold was proposed in this study, which has a simpler preparation process and stronger performance. This study proposes bone scaffolds with an attempt to use polymers that are synthesized separately with three types of minerals as the filler using the microwave foaming method as follows. A 0.1 wt% of montmorillonite (MMT), zinc oxide (ZnO), or titanium dioxide (TiO_2_) is added to chitosan (CS)/gelatin mixtures, respectively, after which sodium bicarbonate is added as a foaming agent, thereby forming porous gels. The polymer synthesized from three minerals was used as filler. The following microwave foaming method was adopted: 0.1 wt% MMT, ZnO, or TiO_2_ was added to the CS/gelatin mixture, and then sodium bicarbonate was added as a foaming agent to form a porous gel. Next, porous gels and polycaprolactone were combined in a self-made mold in order to form bone scaffolds. A stereo microscope is used to observe the morphology of bone scaffolds, after which the pore size analysis, pore connectivity, swell property, porosity, and compressive strength are tested, examining the effects of the mineral type on bone scaffolds. The test results indicate that with MMT being the filler and sodium bicarbonate being the foaming agent, the resulting bone scaffolds yield a porous structure with a pore size between 120 μm and 370 μm. Besides, the incorporation of polycaprolactone also provides samples of 1MCG-P, 2MCG-P, and 5MCG-P with a certain compressive strength of 150–170 MPa. To sum up, the test results substantiate that a combination of the microwave foaming method and MMT generates a porous structure for bone scaffolds (1MCG-P, 2MCG-P, and 5MCG-P), involving a porosity of 38%, an inter-connected porous structure, and the compressive strength that exceeds 150 MPa.

## 1. Introduction

Osseous tissue is a hard dense connective tissue that protects the interior organs and provides the human body support and integrity [1,2]. However, bone tissues could be afflicted with bone injuries and bone defects because of accidents or diseases. Due to considerable damage probabilities, bone tissues are ranked the second most common tissues that receive transplantation, and there is a total of over four million surgeries performed for bone transplants or bone alternative grafts [3,4,5], which suggests that the repair of bone tissues is in high demand and of the essence. Since autogenous bone and allograft tissues far outstrip supply [6], bone scaffolds have come to the fore. Severe bone damage requires surgery to attain reconstruction [7], for which the use of bone transplant substitutes is one measure that helps heal or regenerate bone tissues [8]. Another common clinical problem is the repair of bone defects. Autologous regeneration has its restriction and autogenous bone grafts may trigger transplant rejection. As a result, synthetic bone scaffolds have been a popular approach [9]. 

Bone scaffold refers to the preparation of a kind of cell scaffold with excellent bio-compatibility, which can be gradually degraded and absorbed into the human body. Like natural bone, this bionic scaffold can provide three-dimensional space for cells to survive, so that cells can obtain sufficient nutrients, conduct gas exchange, and eliminate waste. Cells can grow on the scaffold in a prefabricated form and repair bone defects.

A porous structure is a critical feature in the development of bone scaffolds. Chen et al. employed a simple sol–gel approach to producing 3D TiO_2_ foam, and the large size of foam pores subsequently generated large interconnected tunnels [10,11,12]. In light of the studies by Gierszewska et al. and Demir et al., the addition of MMT could improve the properties of CS. Moreover, glutaraldehyde was used to cross-link with CS, during which MMT was added to the resulting cross-link network [13,14]. Hassan et al. compared the CS/siclica and CS/siclica/ZnO nanocomposites in terms of adsorption capacity. The presence of ZnO nanoparticles was proven to enhance the adsorption performance of the nano-composites [15]. 

Polymer foam is relied on for making everything from tissue engineering scaffolds to airplanes. However, traditional polymer foaming methods usually involve chemical reagents or high-pressure gases, which may cause environmental and safety problems. However, the microwave foaming method only uses microwave radiation as the driving force of foaming. Bayrak et al. developed a microwave-induced biomimetic approach [16]. A combination of microwave radiations and gas foaming method generated superporous hydrogel as constituent scaffolds [17,18], after which they were immersed in a 10x SBF solution and processed with microwave treatment at 600 W in order to have an even bony hydroxyapatite coating layer without requiring any other nucleating agent. The study indicated that using the microwave-assisted process to produce bionic HA coating layers could serve as an efficient method in the production of bone scaffolds [19]. Thus, we use NaHCO_3_ as the foaming agent, which is simple and environmentally friendly.

The bone scaffold has a 600 μm pore diameter, 300 μm pore spacing, and orthogonal interconnection between the pores to help ensure sufficient speed into the cavity to maximize cell adhesion and proliferation, and rapid bone tissue ingrowth to improve bone in the body integration; the final total porosity and interconnection path can provide effective vascularization without affecting the mechanical properties of the implant [20,21,22]. Considering the scaffold as the load-bearing implant, the elastic stiffness, strength, and toughness are particularly important. Too much stiffness in the bone scaffolds results in a high-stress shielding phenomenon [23,24]. 

However, sol–gel scaffolds fall short of mechanical strength to compensate for which the gel body is combined with polycaprolactone in this study. For the acquisition of a porous structure, there is substantial literature employing electrostatic spinning, freeze drying, brine leaching, phase separation, and foam gel, but there are few studies employing sodium bicarbonate as the foaming agent. Therefore, this study conducts the microwave foaming method with the aim of sodium bicarbonate as a foaming agent, thereby producing new bone scaffolds. In addition, samples of a gel form that are generated via the microwave foaming method lack mechanical properties, which is addressed by the addition of polycaprolactone afterward. At the same time, three types of minerals, including 0.1 wt% of MMT, ZnO, and TiO_2_, are separately added to the CS/gelatin mixtures, after which sodium bicarbonate (i.e., the foaming agent) and the microwave foaming method are used to make a porous gel. Next, polycaprolactone is combined with the porous gel to produce porous bone scaffolds. The final products are tested for stereomicroscopic observation, pore size measurement, interconnected pore observation, swelling test, and compressive strength test in order to examine the porous structure and mechanical properties of the bone scaffolds. 

## 2. Materials and Methods

### 2.1. Materials

Acetic (Choneye, Taiwan) had a concentration of 99–100%. CS (Sigma-Aldrich, Taufkirchen, Bavaria, Germany) had 85% degree of deacetylation and medium molecular weight. Gelatin was purchased from EMPEROR CHEMICAL Co., Ltd., Taipei, China. Glutaraldehyde (CAS 111308; Alfa Aesar, Stoughton, WI, USA) had a specification of 25% aq. Sodium bicarbonate was purchased from Shimaku Pharmaceutical Co., Ltd., Japan. Natural MMT was purchased from Southern Clay Products, US. TiO_2_ and ZnO were purchased from E. Chang Trading Co., Taiwan, China. Polycaprolactone (Sigma-Aldrich, Taufkirchen, Bavaria, Germany) had a molecular weight of 14,000.

### 2.2. Preparation 

**Figure 1:**
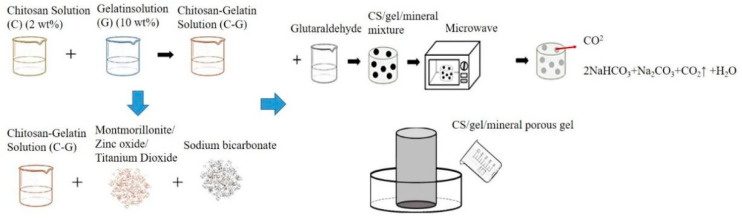
Processing flow chart of CS/gel/mineral porous gel and schematic of self-made mold for bone scaffolds.

Figure 1 shows the procedure for producing porous gel. CS and gelatin mixture was first made as follows. A 2 *v/v*% ethanoic acid and 2 wt% CS solution were heated to 60 °C and simultaneously mixed for twelve hours. CS solution and gelatin solution were blended with a volume ratio of 9:1 and constantly stirred for 4 min at 45 °C. Next, 1 *v/v*% glutaraldehyde (GA) solution was stirred at 25 °C for 5 min, after which 0.1 wt% of MMT, ZnO, or TiO_2_ was added to the CS/gelatin mixture, respectively, followed by 0.02 g of sodium bicarbonate. The mixtures were heated to 55 °C and mixed for 3 min, and then 5 mL of GA solution was incorporated and mixed for 1 min at 55 °C using magnetic stirrers. The final mixtures were kept still at 55 °C on the mixer for another 4 min. The denotations and specifications of samples are listed in Table 1 and Table 2. The final mixtures were then placed in a microwave oven (NN-SM33H, PANASONIC, Fukushima, Osaka, Japan) with an output of 800 W for one minute, and the optimal porous gel was determined accordingly.

Figure 1 shows the procedure for the production of bone scaffolds. The flaky polycaprolactone with a melting point between 50 °C and 64 °C was heated until it became a liquid state. Next, a small amount of Vaseline was smeared over the hollow self-made cylinder mold to decrease the possible difficulty of removing the sample afterward. The liquid was then infused into the mold and not removed until it became solid. The hollow polycaprolactone cylinders were then combined with porous gel, thereby forming the bone scaffolds. Refer to Table 1 and Table 2 for sample definitions and specifications of CS/gel/mineral porous gel and CS/Gel/MMT/PCL bone scaffolds.

#### 2.2.1. Surface Observation 

Bone scaffolds were photographed using a stereo microscope (SMZ-10A, Nikon Instruments Inc., Chiyoda, Tokyo, Japan) with an adjusted focal length and with the aim of ToupView software (Hangzhou ToupTek Photonics, Xihu, Hangzhou, China) in order to acquire images with intensified pixels. 

#### 2.2.2. Pore Size Measurement and Analysis 

ToupView software (Hangzhou ToupTek Photonics, China) was used to measure the pore size, determining the maximal, minimal, and major pore sizes. 

#### 2.2.3. Swelling Property Test 

The swelling property test was conducted based on a modified method according to the study by Ngadaonye et al. [20,25]. Samples were immersed in deionized water at 25 °C and then weighed with an interval of one hour until samples reached the swelling equilibrium status. Three samples for each specification were used.

(1) $$Q=\frac{W_{w}-W_{d}}{W_{d}}$$

where *W_w_* is the sample weight when wet and *W_d_* is the sample weight when dry for a period of time (t).

#### 2.2.4. Compressive Strength Test 

The compressive strength of bone scaffolds was measured using a universal testing machine as specified in ASTM D6641M-09, thereby examining whether they were compatible with the strength required by the impaired bones. The compressive rate was 1.3 mm/min, the distance between two compressive clamps was adjusted to 1.2 cm, and cylinder samples had a diameter of 3.7 cm and a thickness of 1.2 cm. The compressive clamps compress the sample to 50% of its thickness and then undo it. 

#### 2.2.5. Interconnected Pore Measurement

This measurement was designed by the authors. First, 7.5 mL of ethanol and 2.5 g of a black dye were blended to form a coloring solution. The coloring solution was dripped over the surface of samples and left for the evaporation of ethanol. A stereo microscope was used to photograph the surface and cutting section of the samples in order to observe the permeation level, examining whether the samples demonstrate interconnected pores. 

#### 2.2.6. Porosity Measurement 

This test was conducted according to the method proposed by Wu et al. [23,26]. The cell invasion and proliferation were dependent on the porous micropore structure of the stents, which was thus used to measure the porosity in this study. Samples were trimmed into a specified size and immersed in pure water. The height that the water climbs was recorded and computed to have the sample volume and is referred to as *Vv*. The apparent volume was referred to as *V_t_* which includes the solid parts and voids. The porosity can be computed using the equation as follows.

(2) $$Ø=\frac{V_{T}-V_{v}}{V_{T}}\;\;\;\;\;\;\;\;\;\;\;\;\;\;\;\;$$

where Ø is the porosity, is the apparent volume (including solid part and pores), and *V_v_* is the volume of pores. 

#### 2.2.7. Statistical Analysis 

All quantitative data are presented in mean standard deviation using one-way statistical analysis (one-way ANOVA) using THE SPSS Statistics 17 with * *p* < 0.05 and ** *p* < 0.01 indicating the significance level. 

## 3. Results and Discussions

### 3.1. Test Results of Porous Gel

#### 3.1.1. Surface Observation of Porous Gel

**Figure 2:**
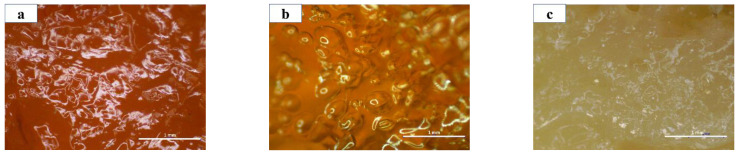
The surface images of CS/gel/mineral porous gel of (**a**) 1MCG; (**b**) 1ZCG; and (**c**) 1TCG.

With the same light source, Figure 2a–c compares different porous gels and 1MCG has a darker shade than 1ZCG and 1TCG, which is primarily because of the dark shade of MMT. By contrast, 1ZCG has a shade that appears white because of the original color of TiO_2_. Despite the shade of the three mineral types, all samples exhibit an even tone, which suggests that the mineral powders can be evenly dispersed in the CS/gel mixtures without causing precipitation or agglomeration. Therefore, the surface observation substantiates that the manufacturing process is stabilized. 

#### 3.1.2. Pore Size of Porous Gel

The data in Table 3 correspond to pore size distributions of 90–160 μm in 1MGC, 100–170 μm in 1ZGC, and 100–140 μm in 1TCG. A previous study indicated that a pore diameter between 50 μm and 710 μm facilitated bone regeneration. It was commonly perceived that a suitable pore size of bone scaffolds was 150–350 μm while a pore size of 200–300 μm fit the growth of cartilaginous tissue [13,27]. Instead of ZnO and TiO_2_, the presence of MMT is more suitable for bone regeneration and the growth of cartilaginous tissue. Furthermore, Peter et al. used a human osteosarcoma cell line (MG-63) in an in vitro experiment and found that cells could adhere to the CS/gelatin stents that were composed of a pore size being 150 μm [25,28], according to which the pore size of our proposed bone scaffolds is adequate. 

#### 3.1.3. Interconnected Pore Analysis of Porous Gel 

**Figure 3:**
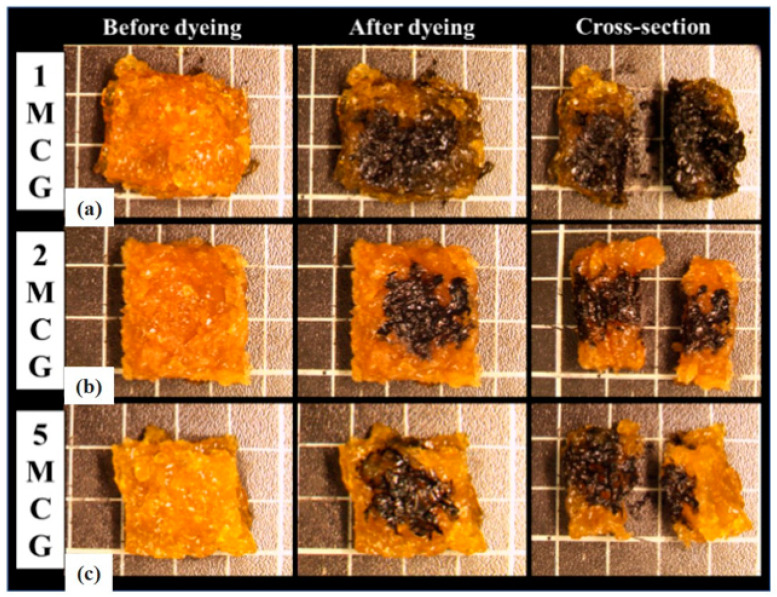
Interconnected pore analysis of CS/gel/mineral porous gel of (**a**) 1MCG, (**b**) 1ZCG, and (**c**) 1TCG.

Bone scaffolds are required to be porous with interconnected pores. A porous feature helps the cell to enter and adhere to the bone scaffolds. The interconnected pores facilitate full cell infiltration inside the bone scaffolds, and the cells obtain the nutrition for growth and proliferation while speeding up the metabolism via the tunnels. Figure 3 shows the cutting section of CS/gel/mineral porous gel (i.e., 1MCG, 1ZCG, and 1TCG) where the dye can be observed. This result indicates that regardless of 1MCG, 1ZCG, and 1TCG, the resulting porous structure has good interconnectivity. 

#### 3.1.4. Swelling Property of Porous Gel

**Figure 4:**
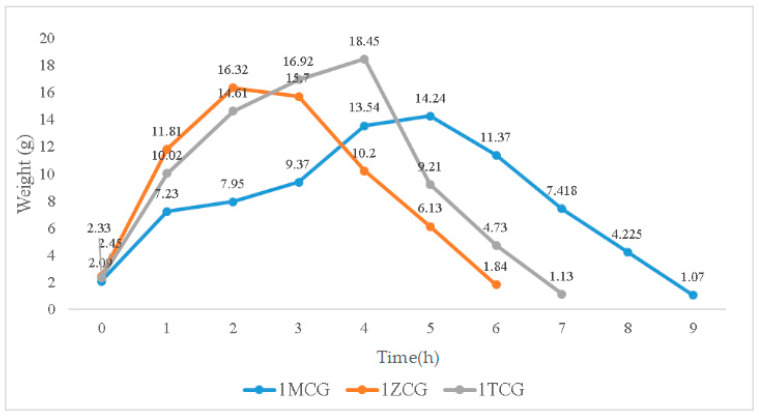
Swelling weight of CS/gel/mineral porous gel as related to the immersion time.

In Figure 4, the swelling weight from 0–0.17 h is used as an initial record because samples are immersed for ten minutes when they start to show a distinctive swelling level. When immersed for 0.17–1 h, the swelling weights of 1MCG, 1ZCG, and 1TCG soar, especially 1ZCG. Conversely, 1MCG exhibits a mitigated raise comparatively. When the immersion time is 1–2 h, the swelling weight of all samples still spikes and 1MCG still remains the lowest rise rate. With an immersion time of 2–3 h, the swelling weight of 1ZCG mildly descends whereas the swelling weight for 1TCG and 1MCG still climbs. With an immersion time of 3–4 h, the swelling weight of 1ZCG drops drastically, but the swelling weight of 1TCG slightly increases, and the swelling weight of 1MCG increases more than that of 1TCG. When the immersion time is 4–5 h, 1TCG has an even lower swelling weight and also starts decomposition, whereas 1MCG still has an increasing swelling weight. With an immersion time of 5–6 h, both 1ZCG and 1TCG are decomposed while 1MCG has a swelling weight that starts descending until the 9th hour when 1MCG is totally decomposed. 

Although 1ZCG and 1TCG have a soaring increase in the swelling weight in the beginning, their swelling weight starts descending in 2 and 4 h, respectively. By contrast, 1MCG does not have a decrease in the swelling weight until the 5th hour of the immersion. Because MMT is composed of single crystals that cannot be bonded compactly, water can have access to the interior easily, which in turn causes MMT to swell. In this study, the three types of proposed porous gels exhibit excellent swell properties, which is not consistent with the findings by Peter et al. In the previous study, the presence of hydroxyapatite in CS/gelatin mixture adversely affected the swell property [25,29]. Conversely, the mineral-containing porous gel exhibits excellent swell properties in this study. 

#### 3.1.5. Porosity Analyses of CS/Gel/Mineral Porous Gel

**Figure 5:**
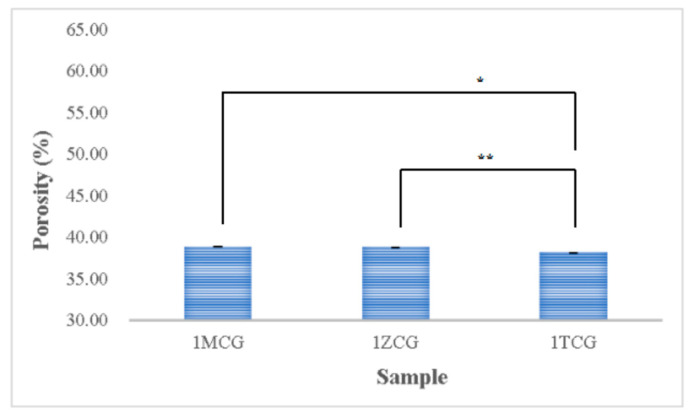
Porosity of CS/gel/mineral porous gel as related to the mineral type (* *p* < 0.05, ** *p* < 0.01).

**Figure 6:**
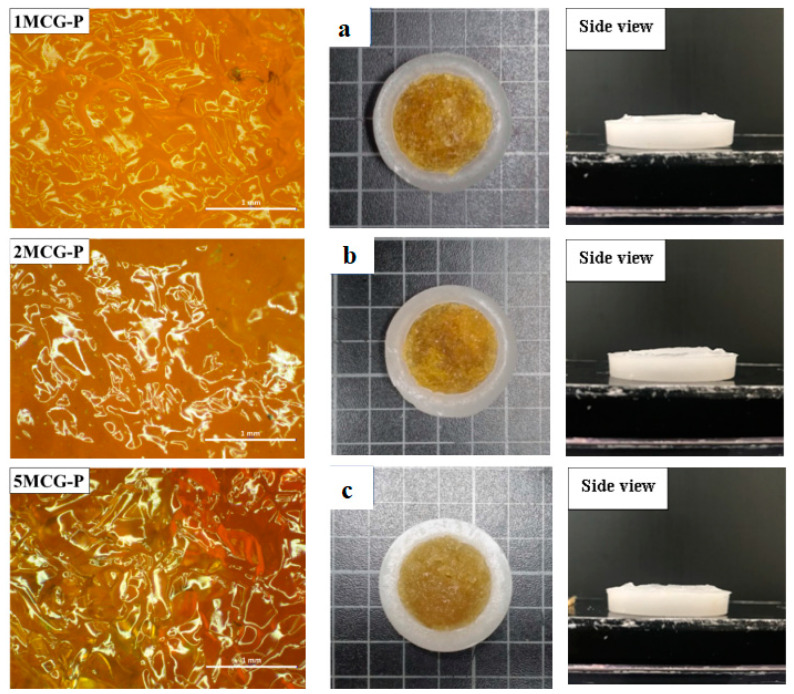
Images of surface of CS/Gel/MMT/PCL bone scaffolds of (**a**) 1MCG-P, (**b**) 2MCG-P, and (**c**) 5MCG-P.

Figure 5 shows that the porosity is 38.854% for both 1MCG and 1ZCG, and 38.174% for 1TCG. In the study by Pandithevan et al., the yielded cortical bone porosity was 40–60% [26,30]. In other words, the three types of CS/gel/mineral porous gels have comparable porosity. Figure 6 compared with Figure 4 indicates the interconnectivity of pores. The comparisons suggest that the porosity is not high but the three types of porous gel all demonstrate a good structure with interconnected pores. In addition, the pore diameter distribution is between 40% and 60%, showing an evenly distributed pore diameter, the results of which demonstrate the same trend that is found in the previous study [26,31].

### 3.2. Test Results of CS/Gel/MMT/PCL Bone Scaffolds

#### 3.2.1. Surface Observation of Bone Scaffolds

**Figure 7:**
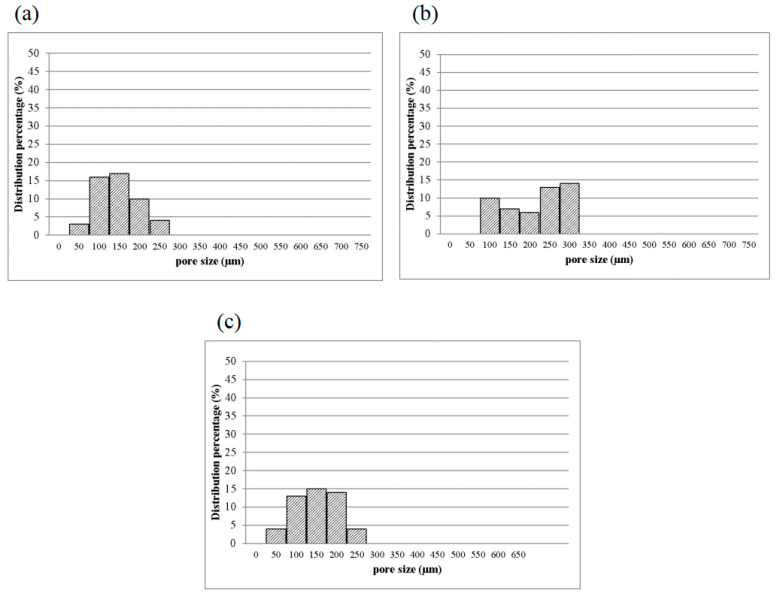
Pore diameter distribution of CS/Gel/MMT/PCL bone scaffolds of (**a**) 1MCG-P, (**b**) 2MCG-P, and (**c**) 5MCG-P.

Figure 6a–c shows that the pore size of 1MCG-P is smaller than that of 2MCG-P or 5MCG-P. Figure 7b also indicates that with 0.2 wt% of MMT, the bone scaffolds acquire denser pores with a diameter that is 0.08–1.4 times that of 1MCG. Figure 5 also corresponds to Figure 6. An increase in the MMT content results in a greater pore diameter because CS and MMT are not sufficiently compatible. Subsequently, MMT-containing bone scaffolds have a comparatively larger size, the result of which is in conformity with the finding by Gierszewska et al. [13,32]. When sodium bicarbonate is in contact with MMT/CS/gelatin mixtures, its interaction with water releases carbon dioxide, which eventually generates a large pore diameter.

#### 3.2.2. Pore Diameter of Bone Scaffolds

Figure 7 shows the pore diameter of bone scaffolds, and the pore diameter is 90–160 μm for 1MCG-P, and 130, 200–270 μm for 2MCG-P, and 150, 320–370 μm for 5MCG-P, so the diameter distribution of the latter appears broad. MMT is composed of multiple layers that are bonded via metal ion bonds and the Van der Waals force. The surface of MMT has negative charges and it constituent layers have the ability to exchange cations that can be inserted and released in appropriate conditions afterward. This particular feature makes MMT an interesting matrix in medicine. In addition, CS is a natural anion polymer that is composed of glucosamine and N-Acetyl glucosamine unit via the β-1-4-glycosidic bond. In a weakly acidic condition, the majority of amino group is rendered with protonation, and the yielded CS thus contains multiple cation electric charges that have a high affinity with the negative-charge layers of MMT [11,33]. Besides, CS is prone to inserting the negative electric charge-containing layers of MMT, which in turn provide the composites with good swell properties, mechanical properties, thermal behavior, and biological adhesion feature.

Turnbull et al. found that bone scaffolds with a pore diameter of 300 μm had a positive influence on bone growth, which was ascribed to the high permeability and high formation of blood vessels. Moreover, a smaller pore diameter that is close to 100 μm facilitates the formation of cartilage, and an increase in the number of pores in the bone scaffolds is substantiated as positive for the formation of blood vessels [3]. Based on the in vitro study by Murphy et al., the yielded pore diameter of 325 μm was helpful to cell attachment, proliferation, and migration [28]. As a result, 2MCG-P which contains 0.2 wt% of MMT obtains an average pore diameter that is 1.63 times that of 1MCG-P and thus has a positive influence on the regeneration of bones and cartilaginous tissues.

#### 3.2.3. Interconnected Pores of CS/Gel/MMT/PCL Bone Scaffolds

**Figure 8:**
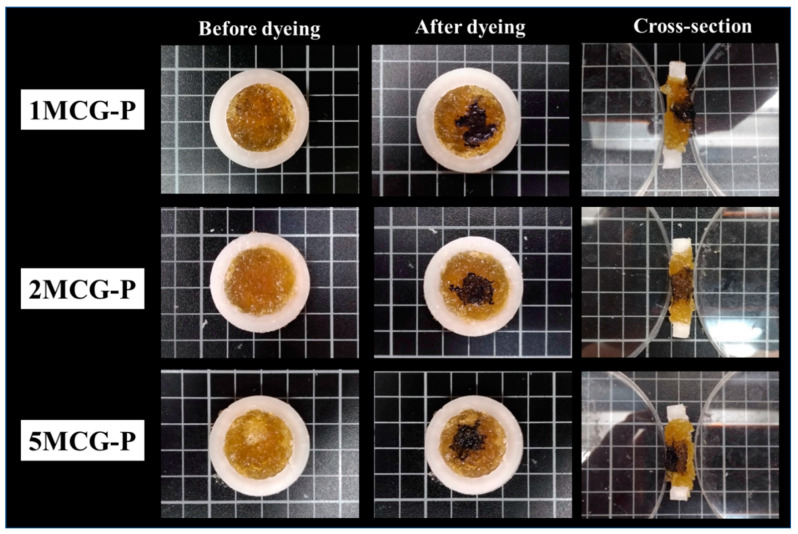
Interconnected pores of CS/Gel/MMT/PCL bone scaffolds.

The interconnectivity of pores is critical for bone scaffolds for only those with interconnected pores can allow cells to enter for a full infiltration and then adhere for proliferation. Besides, the interconnected pores enable the transportation of nutrition and metabolite. Figure 8 shows the pore interconnectivity analyses where cutting sections of 1MCG-P, 2MCG-P, and 5MCG-P exhibit the presence of dye, suggesting that the bone scaffolds have good interconnected pores. Figure 8 also substantiates the infiltration of dye, thereby proving that all of the samples have good interconnectivity.

#### 3.2.4. Swell Property of Bone Scaffolds

**Figure 9:**
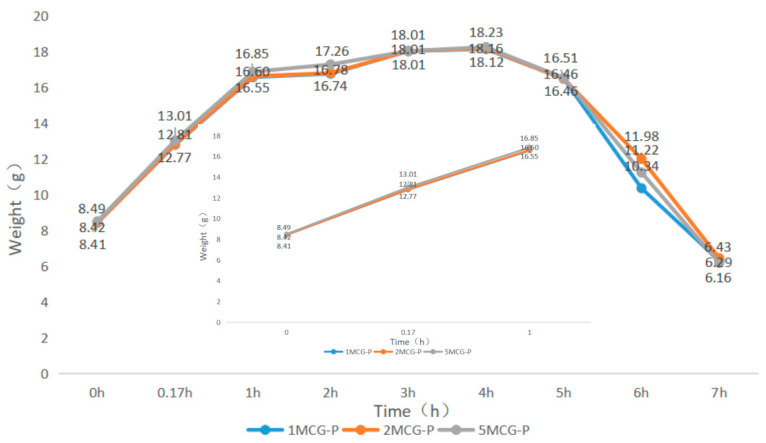
Swell property of CS/Gel/MMT/PCL bone scaffolds.

When bone scaffolds are used in regenerative medicine, the preliminary analyses of degradable materials need to be examined for fluid absorption capacity. The swell property is responsible for the swell performance of polymer matrices, after which the diameter of bone scaffolds is increased, which facilitates cell adhesion and internalization, which is imperative to improve the regenerative process [29]. In Figure 9, an immersion time of 0–0.17 h is set as the initial test time point because moisture-absorbent materials exhibit a significant swelling performance after being immersed for ten minutes (cf. the inset of Figure 9). A 1-h immersion speeds up the increase in weight of 5MCG and likewise, a 2- and 3-h immersion leads to a significant increase in the weight for 2MCG-P. With an immersion time of 3–4 h, 1MCG-P, 2MCG-P, and 3MCG-P show a comparable increase in wet weight. 1MCG-P, 2MCG-P, and 5MCG-P start to have flaking edges which in turn reduce the wet weight after being immersed for 4 h, and they show a remarkable decrease in the weight after a 5-h immersion, and finally have a lower wet weight than the dry weight after a 7-h immersion because gelatin is slightly dissolved in water.

#### 3.2.5. Porosity Measurement of CS/Gel/MMT/PCL Bone Scaffolds

**Figure 10:**
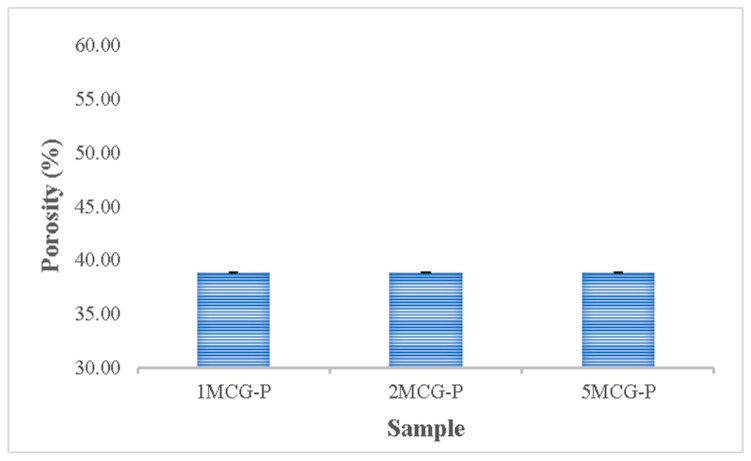
Porosity of CS/Gel/MMT/PCL bone scaffolds (without significant difference).

The porosity of bones indicates the volume taken by those constituent voids in bones. Pandithevan et al. found that cortical bone porosity was 40–60% [26]. 1MCG-P, 2MCG-P, and 5MCG-P have a comparable porosity of 40–60%. Compared with Figure 10, the proposed bone scaffolds have a normal porosity level as well as a porous structure with interconnected pores. This result indicates that this study manufactures bone scaffolds with a required pore size range and an even pore diameter distribution. Moreover, the statistical analyses also indicate that there is no significant difference in the porosity among all samples, and the average porosity is 38.854%.

#### 3.2.6. Compressive Strength of Bone Scaffolds

**Figure 11:**
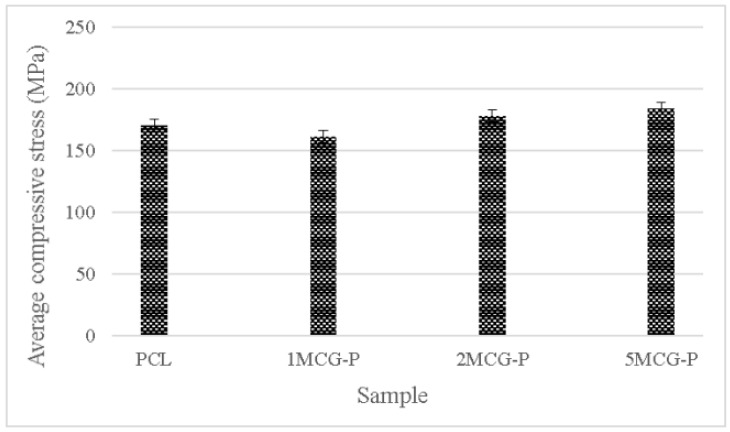
Compressive strength of CS/Gel/MMT/PCL bone scaffolds.

To sum up, CS is a natural biopolymer with widespread availability. Its absorption, biodegradability, and biocompatibility make it ideal for extensive application [30], the proposed porous gel consists of three minerals (i.e., MMT, ZnO, and TiO_2_) individually, and the group containing MMT outperforms the other two groups. Therefore, CS/Gel/MMT porous gel with constitutional 0.1 wt%, 0.2 wt%, and 0.5 wt% is used for further comparison. Because porous gel has lower strength, in compensation polycaprolactone is incorporated to form bone scaffolds. The compressive strength test is conducted afterward. Polycaprolactone is commonly used for reinforcement in order to simulate the mechanical strength of ECM polymer as seen in the study by Wang et al. [31]. Different reinforcing methods provided bone scaffolds with different compressive strength levels. For example, Liu et al. combined polycaprolactone and collagen to form a slurry, from which the solvent was then removed, thereby forming hollow stents with the compressive strength being 0.5–2.0 MPa. Moreover, a 3D printing technique could form stents with a compressive strength of 3–6 MPa [32]. In past research, CS also has considerable potential in the biomedical field for solution casting. Comparatively, in past research, CS also has considerable potential in the biomedical field for solution casting [33], strengthening polycaprolactone via the melt-casting mold method improves proposed bone scaffolds in terms of compressive strength. Furthermore, the compressive modulus is highly dependent on the microstructure of matrices. A previous study indicated that the compressive modulus was inversely proportional to porosity and pore diameter. By contrast, an increase in the pore thickness or a decrease in porosity had a positive influence on the compressive modulus [34]. Figure 11 shows that the average compressive strength is 170.207 MPa for polycaprolactone (PCL), 161.289 MPa for 1MCG-P, 178.239 MPa for 2MCG-P, and 183.966 MPa for 5MCG-P. Samples can be ranked from lowest to highest according to the average compressive stress as 1MCG-P, 3MCG-P, and 5MCG-P. Because the difference in porosity is rather small, the compressive stress increases as a result of increasing pore diameter. Coe et al. reported that compressive strength is 150 MPa for compact bones, 4–15 MPa for spongy bone, 161.289 MPa for 1MCG-P, 178.239 MPa for 2MCG-P, and 183.966 MPa for 5MCG-P. The proposed bone scaffolds in this study have comparable compressive strength with compact bones [35,36,37].

## 4. Conclusions

In this study, the microwave foaming method is employed to produce porous bone scaffolds. CS and gelatin are mixed, after which MMT, ZnO, or TiO_2_ is added to the mixture and blended in order to form a porous gel. The gel is then combined with PCL in order to form compression-resistant porous bone scaffolds. The authors designed the pore interconnectivity measurement that can successfully observe and evaluate whether bone scaffolds have interconnected pores. The 5MCG-P group containing 0.5 wt% of MMT has a porosity rate that is 48% greater than that of the 1MCG-P group containing 0.1 wt% of MMT. In addition, the proposed bone scaffolds of 1MCG-P, 2MCG-P, and 5MCG-P yield a comparable porosity with that of cortical bones which is 40–60%. The pore diameter increases as a result of a rise in the MMT content. The maximal compressive stress is 227.013 MPa with an average between 161 MPa and 183 MPa, and the compressive strength of 5MCG-P is 1.14% higher than that of 1MCG-P. This study employs the microwave foaming method that has been rarely used in previous studies and combines porous gels with PCL in order to produce porous, compression-resistant bone scaffolds. The innovative manufacturing process is capable of producing bone scaffolds that have pore diameters as expected.

## Figures and Tables

**Table 1:** Denotation and specification of CS/gel/mineral porous gel.

Denotation	Mineral		Chitosan (mL)	Gelatin (mL)	GA (mL)	Sodium Bicarbonate (g)
	Type	Amount				
1MCG	MMT	0.025	9	1	2.5	0.01
1ZCG	ZnO	0.025	9	1	2.5	0.01
1TCG	TiO_2_	0.025	9	1	2.5	0.01

**Table 2:** Denotation and specification of CS/Gel/MMT/PCL bone scaffolds.

Denotation	MMT Content (g)	Chitosan (mL)	Gelatin (mL)	GA (mL)	Sodium Bicarbonate (g)
1MCG-P	0.025	9	1	2.5	0.01
2MCG-P	0.050	9	1	2.5	0.01
5MCG-P	0.125	9	1	2.5	0.01

**Table 3:** Pore diameter of CS/gel/mineral porous gel.

Sample Type	Minimum Pore Size (μm)	Maximal Pore Size (μm)	Average Pore Size (μm)	Primary Pore Size Distribution (μm)
1MCG	50	250	150	90–160
1ZCG	100	280	190	100–170
1TCG	120	240	180	100–140

## Data Availability

No report data.
